# Re-evaluating hereditary breast and ovarian cancer risk: clinical impact of updated multigene panel sequencing and genetic counseling

**DOI:** 10.1007/s10689-026-00547-2

**Published:** 2026-03-27

**Authors:** Julie Isabelle Plougmann Gislinge, Anna Byrjalsen, Klara Vinsand Naver, Helle Vibeke Clausen, Pernille Ravn, Kresten Rubeck Petersen, Karin A. W. Wadt

**Affiliations:** 1 Department of Gynecology, Obstetrics and Fertility, Copenhagen University Hospital Herlev, Borgmester Ib Juuls Vej 1, 2730 Herlev, Denmark; 2 Department of Clinical Genetics, Copenhagen University Hospital, Rigshospitalet, Blegdamsvej 9, 2100 Copenhagen, Denmark; 3 Department of Gynecology, Obstetrics and Fertility, University of Southern Denmark Odense Hospital, J. B. Winsløws Vej 4, 5000 Odense, Denmark

**Keywords:** Breast cancer, Ovarian cancer, Hereditary, Next-generation sequencing, Pathogenic variant, Surveillance

## Abstract

An increased lifetime risk of ovarian cancer is observed among women with a family history of ovarian cancer, with or without breast cancer, as well as among carriers of pathogenic germline variants in ovarian cancer (OC) predisposition genes. Over the past two decades, additional OC–associated genes beyond *BRCA1/2* have been identified, highlighting the need for updated genetic evaluation in women previously classified as high-risk without a known PV to ensure correct diagnosis and prevent overtreatment. This retrospective, quality assurance cohort study aimed to evaluate whether updated multigene panel sequencing and genetic counseling improve risk stratification. All women enrolled in the OC surveillance program at Copenhagen University Hospital Herlev between 2018 and 2023 and received counseling prior to the implementation of the revised 2017 guidelines in Denmark, were included. Among 674 women (median age 40.9), 174 had a *BRCA1* PV, 168 had a *BRCA2* PV, 55 had PVs in moderate-risk genes, and 277 were enrolled based on family history of OC ± BC alone. Of these 277, 216 (78%) underwent updated genetic testing and counselling; 165 (76%) had no increased risk of OC and were released from gynecological surveillance. Over a mean follow-up of 48 months, none developed OC, and one developed BC, corresponding to a negative predictive value of 99% for the updated risk assessment. Updated genetic testing and counseling significantly improve risk stratification in women previously classified as increased risk of OC based solely on family history. This supports re-evaluation of at-risk women and families to guide appropriate surveillance, reduce unnecessary follow-up and interventions, and align clinical practice with evolving guidelines.

**Supplementary Information:**

The online version contains supplementary material available at 10.1007/s10689-026-00547-2.

## Introduction

Genetic counseling of families at increased risk of breast and ovarian cancer has evolved significantly since being introduced around 2000. In Denmark, a major shift occurred in 2018 [1], when the approach to counseling became more individualized, with assessments of risk for breast or ovarian cancer conducted separately. The term hereditary breast and ovarian cancer (HBOC) is now only used when a *BRCA1* or *BRCA2* pathogenic variant (PV) is identified. Previously, women with a family history of both breast and ovarian cancer, but without a PV in *BRCA1/2,* was given the diagnosis “HBOC without a PV”. Subsequently, they were referred to gynecological surveillance, and many women continued to undergo surveillance based on older assessments. Following reclassification based on new guidelines, many of these women are now categorized as having no elevated cancer risk. HBOC is currently defined by a pathogenic germline variant in *BRCA1/BRCA2* genes and carriers have 20‒50% lifetime risk of developing ovarian cancer (OC) compared to a risk of 1‒2% in the general population [2, 3]. The definition has previously been unclear, but in 2019 in Utah, USA it was estimated that around 12% of women under surveillance for HBOC have only a familial disposition with no PV, while the majority had a determined genetic predisposition [4].

Surveillance for hereditary ovarian cancer (HOC) with annual transvaginal ultrasonography (TVUL) and evaluation of serum tumor marker Ca-125 is considered to have a high specificity, but a low positive predictive value (PPV), especially in early-stage disease [5, 6]. Some studies have shown surveillance to increase risk of complications due to false positive results with subsequent unnecessary surgery without mortality reduction. Therefore, correctly diagnosing HBOC or HOC is important, so risk reducing surgery can be offered at appropriate age [7]. Risk-Reducing Bilateral Salpingo-Oophorectomy (RRSO), either with or without hysterectomy, reduces the risk of OC with about 90% [8–10] *and* is the gold standard for reducing OC risk in women with a hereditary predisposition. Surgery recommendations are age dependent—for women with *BRCA1* age 35‒40 years, *BRCA2* 40‒45 years and women without a PV, but with a family history of multiple cases of OC no later than 50 years of age [7]. This differentiation in recommendation is due to the different age dependent penetrance of the possible OC based on PV and/or family history, which highlights the need of comprehensive genetic testing and counselling to ensure the best possible treatment, while preventing overtreatment) [3, 7]. The use of a extended Next Generation Sequencing (NGS) gene panel has contributed to update genetic counselling in this population, where moderate OC penetrance variants in genes such as *RAD51C/D and BRIP1* have been included [12–14]. Whereas *BRCA1/BRCA2* are high penetrance genes, conferring a high lifetime risk of OC, other genes confer a moderate risk of developing OC [7]. Recent clinical guidelines have suggested how several genes can be categorized based on the lifetime risk of developing OC compared to the general population. Furthermore, a recent consensus statement from the UK recommends that surgery should be offered to women with a residual risk of 4–5% or more [3, 7,11].

The ability to prevent cases of hereditary related OC has been investigated in nationwide cohort studies. Wide genomic testing has been shown to reduce cancer cases, increase the number of quality adjusted life years (QALYs) and to be cost-effective in terms of medical costs vs. the cost of genomic testing [15], indicating genetic screening and eventually RRSO in women with HBOC/HOC to herald substantial overall benefits regarding morbidity, mortality, and overall quality of life. Updated genetic testing can establish the risk of developing OC more precisely [7], which could potentially save lives by identifying women at high risk of OC and offer them a timely RRSO, and spare women at low risk of OC a lifetime of check-ups, anxiety of developing cancer, unnecessary surgery, and subsequent hormone replacement therapy (HRT).

Our aim was therefore to investigate if extended genetic screening with NGS and updated genetic counselling improves the diagnostic accuracy in women previously diagnosed with increased risk of OC based on family history.

## Method

We performed a retrospective, double-center quality assurance cohort study. All genetic counselling was performed at the Department of Clinical Genetics, Copenhagen University Hospital, Rigshospitalet; Genetic screening was performed at Genomic Medicine, Rigshospitalet; and all gynecological surveillance and subsequent surgery were performed at the Department of Gynecology, Obstetrics and Fertility, Copenhagen University Hospital Herlev.

### Inclusion criteria

- All women enrolled in the surveillance program for HBOC/HOC at the Department of Gynecology, Obstetrics and Fertility, Herlev Hospital from 2018 to 2023, with the following diagnostic codes (WHO ICD-10)
  - DZ803 (family history of breast cancer)
  - DZ804 (family history of cancer in the reproductive system)
  - DZ848A (family history with a known genetic defect)
  - DZ848A2 (family history with hereditary breast- and ovarian cancer (HBOC))
  - DZ809 (family history with cancer without further specification)

### Exclusion criteria

- Incorrect diagnosis
- Never evaluated at either the Department of Clinical Genetics, Rigshospitalet or at the Department of Gynecology, Obstetrics and Fertility, Herlev Hospital
- If the woman had declined to participate in quality assurance studies

**Fig. 1:**
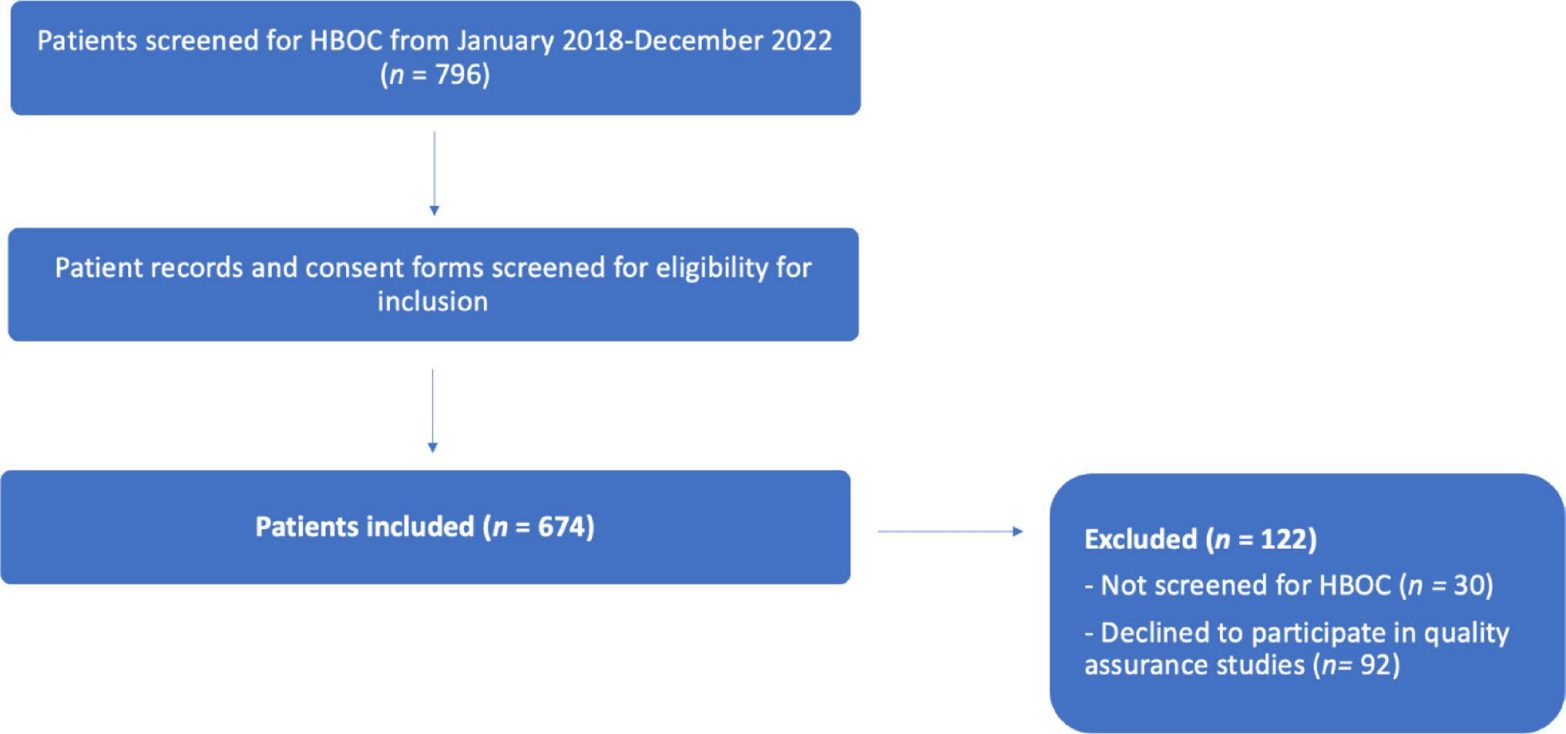
Flowchart of the process of patient inclusion

796 women were evaluated for inclusion and in total n = 674 women were included (Fig. 1).

Patient data, including results of the genetic analysis, were extracted from patient files in our regional electronic health platform, *EPIC Sundhedsplatformen*. Pathology reports following risk reducing surgery, biopsies or specimens from cancer, if present, were extracted from the regional pathology database, *PatoBank*.

### Outcomes

Our primary outcome was to evaluate the effect of updated genetic screening and counselling on risk stratification for OC.

Secondary aims were:

1. To evaluate the genetic landscape and to identify pathogenic variants in moderate risk genes amongst women screened for HOC/HBOC
2. To assess how many women were spared unnecessary surveillance and surgery

### Genetic analysis

DNA was extracted from peripheral blood cells, and a NGS gene panel was performed (see Supplementary Table 1). All PVs were evaluated in a clinical setting by a senior doctor in clinical genetics and was curated according to gene specific ACMG classification. The BOADICEA risk model were also applied to further asses the risk of breast and ovarian cancer separately [16]. Women from families with a known PV or low risk pathogenic variant (LPV) in *BRCA1/2* were offered presymptomatic testing for the family variant according to guidelines. In families were the index patient (OC patient) did not have a PV in one of the five high risk genes (*BRCA1/2, TP53, PTEN, CDH1*) that was tested for prior to the 2019 guidelines, no additional testing of the referred patient were performed, and she was therefore categorized as “HBOC without a known PV”.

### Statistical analysis

Baseline characteristics of the patient population and follow-up period is summarized as means (with standard deviation) and medians (with interquartile range) for continuous data and as frequencies for categorical data. For comparison of categorical data, *chi-square* or *Fisher’s exact tests* was used when appropriate, whereas *students t-test* or *One-way ANOVA* was used for continuous data. Sensitivity, specificity, positive predictive value (PPV) and negative predictive value (NPV) was calculated when appropriate. Statistical significance is determined at p < 0.05. For all statistics, IBM SPSS Statistics version 25 was used.

## Results

674 women enrolled in our gynecological surveillance program were included in this study. Mean age at inclusion were 40.9 years (range 18‒83), for patient characteristics see Table 2. 174 women (25.8%) carried a germline PV in *BRCA1*; 168 (24.9%) carried a germline PV in *BRCA2*; 51 (7.6%) had Lynch syndrome or PVs in other OC predisposing genes; four (0.6%) did not carry pathogenic *BRCA1/2* variants but heralded from a *BRCA1/2* positive family with accumulated cases of BC and OC; and finally, 277 (41.1%) had no known PVs, but had a reported family history of OC ± BC, and was therefore previously recommended to undergo gynecological surveillance. 146 women (21.7%) had BC before initiating gynecological surveillance, while no women had OC before initiating gynecological surveillance.

**Table 2:** Demographics and patients characteristics. IQR = interquartile range

Age, inclusion in the gynecological HBOC surveillance (mean, range)	40.9 (18‒83)
Age at menopause (natural, medical and surgical) (mean, range)	46.1 (31‒60)
Reason for surveillance (n, %)	BRCA1: n = 174 (25.8%) BRCA2: n = 168 (24.9%) Another PV incl. Lynch syndrome: n = 51 (7.6%) BRCA negative from a BRCA positive family with accumulated cases of BC/OC = 4 (0.6%) Family history: n = 277 (41.1%)
Risk-reducing surgeries (n, %)	Risk reducing mastectomy: n = 156 (23.1%) Risk reducing salpingo-oophorectomy (RRSO): n = 246 (36.5%) + hysterectomy): n = 13
Length of follow-up (in months, median, IQR)	48 (IQR 25% 14, IQR 75% 88.25)

### Updated genetic screening and counselling

**Fig. 2:**
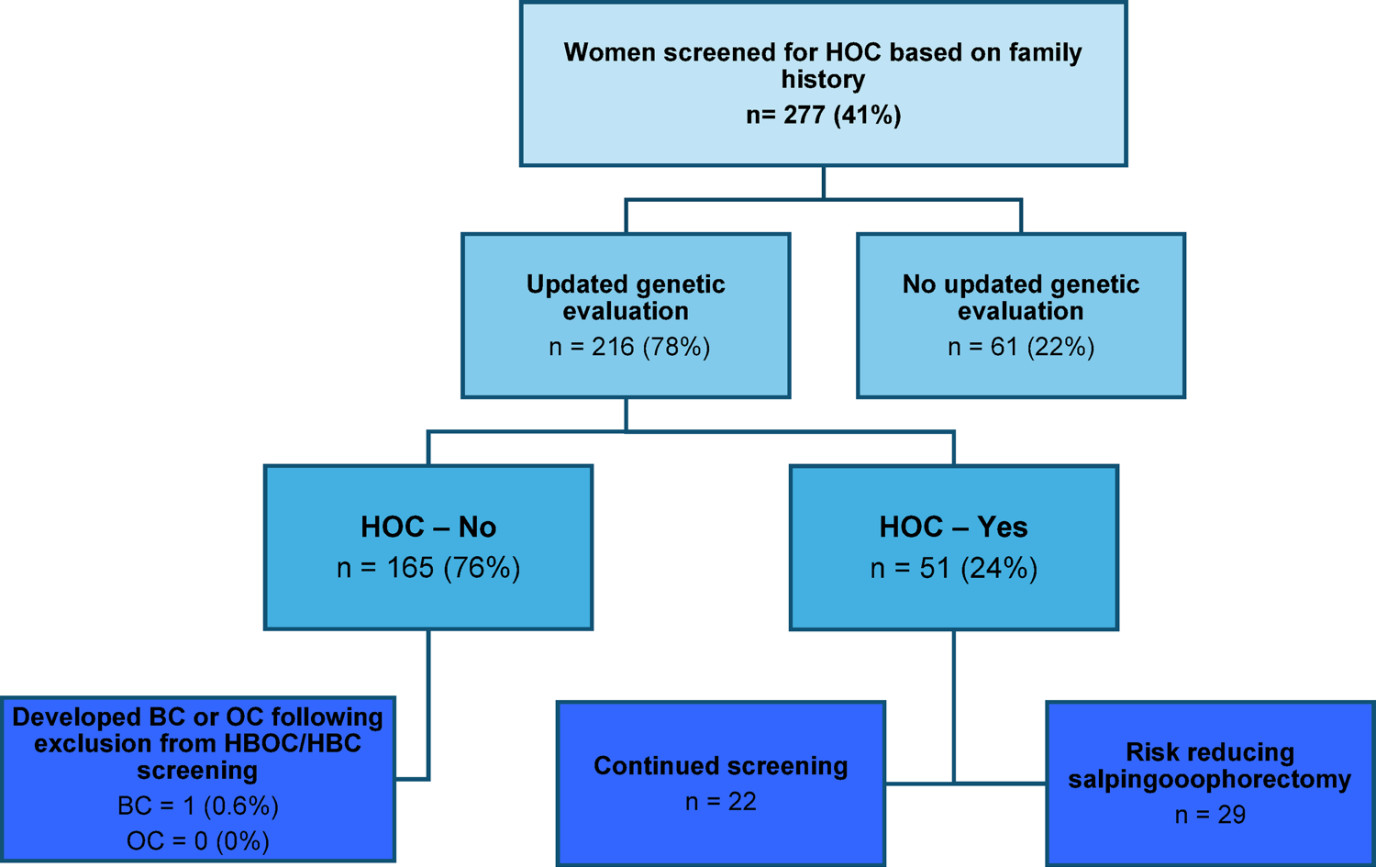
Outcome of extended genetic screening and genetic counselling of patients initially included in the surveillance program based on family history. HBOC = hereditary breast- and ovarian cancer; HOC = Hereditary ovarian cancer; HBC = Hereditary breast cancer

365 (54.2%) women underwent updated genetic screening with NGS counselling, either due to a diagnosis of BC or OC (n = 149) or as part of an updated risk estimation due to prior surveillance (n = 216). Of the 216 women re-evaluated, 165 (76.4%) had no increased risk and was excluded from the gynecological surveillance program. 51 (23.6%) were still at high risk for OC based on a newly discovered PV (6 women, 11%. See Table 3) or a continued high risk without a PV based on family history (45 women, 88.2%) and either continued surveillance (n = 22) or underwent RRSO (n = 29) (Fig. 2). 61 women (22%) who underwent surveillance due to family history of cancer did not undergo updated genetic screening and counselling due to various reasons (see supplementary Table 2).

**Table 3:** Overview of pathogenic variants found in the entire cohort (left, n = 365) and the subgroup screened based on family history (right, n = 216)

Pathogenic variants discovered with NGS (all screened) (n = 130)	BRCA1 = 56 BRCA1 (1699Q) = 1 BRCA2 = 53 BRIP1 = 4 MLH1 = 2 MSH2 = 1 MSH6 = 2 PALB2 = 4 PMS2 = 1 RAD51C = 4 RAD51D = 2	Pathogenic variants discovered with NGS (familiar history) (n = 6)	BRCA1 = 1 BRCA2 = 2 MLH1 = 1 RAD51D = 1 PMS2 = 1

Of the 165 women excluded from the gynecological surveillance program due to low risk of OC (< 4% lifetime risk), 11 (5.1%) developed cancer. Six women developed BC, and one was a woman, who had already had ductal carcinoma in situ at the same breast before inclusion in the surveillance program. Of the six, five were diagnosed while enrolled in the breast surveillance program due to increased risk of BC, but were excluded from gynecological surveillance due to no increased risk of OC. The remaining five cancers were one cholangiocarcinoma; one malignant glioma; one ventricular GIST tumor; one malignant melanoma; and one basocellular carcinoma.

### Quality assessment of the updated genetic screening and counselling

Sensitivity, specificity, PPV and NPV of the updated genetic screening and counselling for the diagnosis of HOC/HBOC was 38.5%, 49.3%, 5.5% and 91.3%, respectively. If adjusting for the five women that developed BC following exclusion from the gynecological surveillance program due to low risk of OC but were still following breast cancer surveillance due to a high risk for BC, the negative predictive value for the updated genetic screening and counselling for OC/BC reaches 99%.

### Pathogenic genetic variants discovered with NGS

Of the 277 women, who were screened based on family history alone, 216 (78%) ended up undergoing updated genetic evaluation. Six (2.7%) carried a PV and 37 (17.1%) carried one or more variants of unknown significance (VUS) (Table 3, supplementary Table 2 and supplementary Fig. 1a–d). Of the total 365 women evaluated with NGS, including those evaluated due to a diagnosis of breast cancer (n = 149), 130 (35.6%) had a PV. Of those, only six women screened based on family history had a PV identified in the NGS gene panel (Table 3). The most frequent PV were identified in *BRCA1* (43%) and *BRCA2* (41%) followed by *BRIP1* (3%), *PALB2* (3%) and *RAD51C* (3%) and *RAD51D* (1.5%).

### Follow-up

Follow-up was determined from entering the gynecological surveillance program until inclusion in our cohort, or if patients moved to other parts of Denmark or abroad, as the latest entry in their electronic medical record, *EPIC* *Sundhedsplatformen*. There was a statistically significant difference in follow-up length for women with other pathogenic variants having the shortest follow-up (mean 27.36 months) and women with a family history having the longest follow-up (mean 74.4 months) (*p* = *0.015*)*.* 28 women (4.2%) developed BC or ductal carcinoma in situ (DCIS) while enrolled in the surveillance program (nine DCIS, 19 BC). Four women (1.6%) of the 246 who underwent RRSO had premalignant or malignant lesions in their uterine tubes or ovaries (two tubal cancers, three serous tubal intraepithelial carcinoma (STIC) lesions,), and two (0.8%) developed peritoneal cancer following RRSO – both *BRCA* carriers.

## Discussion

In this study, we included 674 women at increased risk of OC due to a germline PV in *BRCA1/2*, a PV in a moderate-risk gene, or a family history of OC and/or BC. Of these, 277 women (41.1%) were initially enrolled in the surveillance program based on family history alone—a notably higher rate than reported in other countries (~ 12%) [4]. This finding indicates that a higher proportion of women in our cohort were screened for HBOC without a known PV compared with previous studies. Among the 277 women enrolled based on family history alone, 216 (78%) underwent updated genetic testing and counselling. Following re-evaluation, 165 (76%) were found not to have an increased risk of OC and were subsequently excluded from the gynecological surveillance program, reducing the proportion of women screened based on family history alone to 24%. Only six women (2.7%) were identified with a PV, which is lower than reported in other studies [17–20]. However, when including variants of uncertain significance (VUS), the combined detection rate was 19.8%, which is concordant with findings from Germany in 2023 [17] and Hungary in 2024 [20]. The distribution of *BRCA1/BRCA2* also aligns with Canadian data from 2020 [18].

The higher proportion of VUS compared with PVs likely reflects the composition of our cohort, in which broader criteria were previously used to identify women at increased risk of OC, and this approach differs from other international studies. Additionally our clinically validated genetic testing panel included 46 genes relevant to hereditary breast and ovarian cancer risk genes, whereas the German study analyzed 123, which may explain the lower PV detection rate. Another contributing factor may be that many women were enrolled in surveillance prior to the updated 2019 guidelines and would not have met criteria for genetic testing if referred later. This is likely to explain why 76% of women were reclassified following updated genetic testing and counseling and were consequently released from surveillance.

During a mean follow-up of 48 months, none of the women excluded from gynecological surveillance developed OC, and only one woman developed BC. This resulted in a NPV at 99% for HOC/HBOC, when updated genetic screening and counselling with risk calculation, did not indicate an increased risk. This high NPV provides supportive evidence for the safety of updated risk stratification, especially when excluding women from a surveillance program that some have followed for years. However, our findings are limited by a relatively short follow-up and the cohort’s mean age of 41 years, as OC rarely develops at younger ages, even among women with a hereditary risk due to a PV.

This study highlights the importance of continued collaboration between genetic and clinical departments managing women at increased cancer risk. Genetic knowledge and testing capabilities have improved considerably over the past decade; however, these advantages has primarily benefitted newly diagnosed patients with BC diagnosed after age 50 or with OC, who are routinely offered to undergo genetic counselling and testing. Families previously assessed under earlier genetic testing paradigms have often been followed based on an outdated risk estimation, and our findings demonstrate that systematic re-evaluation and updated testing allow for a more accurate risk stratification and subsequent clinical follow-up. Expanded genetic testing also aids in identification of other PVs, which may lead to either intensified surveillance (e.g., *Lynch syndrome*), or reduced surveillance (*RAD51C*). Furthermore, women with a residual lifetime risk of OC < 5% are no longer considered at substantially increased risk and are now only recommended surveillance based on CANRISK evaluation (https://www.canrisk.org/), in accordance with national surveillance guidelines. The CANRISK tool combine family history and genetic data and is used clinically to evaluate the risk of breast and/ or ovarian cancer.

Previous studies have shown socio-economic benefits of timely and accurate diagnosis of HOC/HBOC. Expanded genetic testing have been shown to reduce the incidence of HBOC related cancers, increase quality-adjusted life years (QALYs), and be cost-effective in terms of lifetime medical costs vs. the cost of genomic testing, in both pre- and postmenopausal women [21–23]. Additionally, women undergoing genetic testing are more likely to undergo risk reducing mastectomy (RRM) and/or RRSO, significantly decreasing their lifetime risk of developing HBOC related cancers [24]. Although gynecological surveillance has limited efficacy in detecting early-stage disease [5, 6], it remains valuable in facilitating informed decision-making about the timing of RRSO. In Denmark, women are counseled at their first gynecological visit that surveillance is not the gold standard for OC prevention, and that RRSO provides the most effective risk reduction. However, some women find it difficult to decide when—or whether—to undergo surgery and need time to process their diagnosis and adapt to living a “cancer surveillance life” [25]. Therefore, annual og bi-annual follow-up remains essential, particularly for women who delay RRSO beyond the recommended timeframe {Clausen HV. et al., 2017}.

In settings where genetic and gynecological resources must be carefully allocated and where overdiagnosis and excessive treatment occur, prioritizing and correctly diagnosing patients with hereditary cancer syndromes is crucial [26, 27]. This study reflects a real-world re-evaluation of clinical practice following guideline updates rather than a prospective screening intervention Updated genetic evaluation allowed 165 women to be safely released from surveillance, thus freeing clinical capacity within gynecological departments for patients with greater need and reducing waiting time for counselling and treatment. Additionally, unnecessary surgeries—and their associated physical, psychological, and sexual complications—were avoided [28, 29]. Finally, women undergoing surveillance based solely on family history had the longest follow-up of all the groups with a mean of 74.47 months (*p* = *0.015*). With updated genetic testing and counselling, these women can now receive a correct diagnosis prior to initiation of gynecological surveillance, reducing the number of women living a “cancer surveillance life” with the associated difficulties and ambivalences that follows [25]. We will continue to monitor this cohort to assess the long-term effect of the updated genetic counselling. Our findings underscore the importance of continuous re-evaluation of families at increased cancer risk using evolving genetic knowledge to support accurate risk assessment and appropriate long-term care.

## Conclusion

Continued collaboration between genetic and clinical departments in the management of women with an increased risk of OC is essential to ensure follow-up according to current guidelines. Re-evaluation is crucial for women without a PV associated with an elevated OC risk. Updated genetic testing and counselling demonstrated a high negative predictive value (NPV) of 99% for both OC and BC. Although our follow-up period is relatively short, our findings support this screening and counselling approach as standard practice for women referred for gynecological surveillance—and should be implemented wherever it is not yet applied. This ensures that women with a genuinely high risk of BC and/or OC continue appropriate surveillance and timely risk-reducing surgery, while those without increased risk are spared prolonged medical surveillance, cancer-related anxiety, and potentially unnecessary life-altering surgeries.

## Supplementary Information

Below is the link to the electronic supplementary material.

Supplementary Material 1

## Data Availability

No datasets were generated or analysed during the current study.
