# Supplementary material for: Re-evaluating hereditary breast and ovarian cancer risk: clinical impact of updated multigene panel sequencing and genetic counseling
Source: Fam Cancer. 2026 Mar 27;25(2):35. doi: 10.1007/s10689-026-00547-2 (PMC13031182; doi:10.1007/s10689-026-00547-2)
Supplement: Supplementary file 1 — Supplementary Material 1 [file 10689_2026_547_MOESM1_ESM.docx]

## Supplementary files

**Supplementary table 1:** Gene panel including 46 genes used for the updated genetic screening and counselling

| **ACD**  **APC**  **ATM**  **AXIN2** | **BAP1**  **BARD1**  **BMPR1A**  **BRCA1**  **BRCA2**  **BRIP1** | **CDH1**  **CHEK2**  **CDK4**  **CDKN2A**  **CDKN2B** | **DICER1** |
| --- | --- | --- | --- |
| **EPCAM** | **FH**  **FLCN** | **GREM1** | **MET**  **MLH1**  **MLH3**  **MSH2**  **MSH3**  **MSH6**  **MUTYH** |
| **NTHL1** | **PALB2**  **PMS2**  **POLD1**  **POLE**  **POT1**  **PTEN** | **RAD51C**  **RAD51D**  **RNF43**  **RPS20** | **SDHB**  **SDHC**  **SMAD4**  **STK11** |
| **TERF2IP**  **TERT**  **TP53** | **VHL** |  |  |

**Supplementary table 2:** Reasons for not receiving updated genetic surveillance and counselling

| Women, who did not receive updated genetic evaluation (n = 61) |  |
| --- | --- |
| Underwent risk-reducing surgery before 2019 (new guidelines) | *n = 15* |
| Declined updated genetic evaluation | *n = 11* |
| Family member was genetically evaluated | *n = 8* |
| Other reasons | *n = 7* |
| Negative for BRCA1/2, but assessed as high risk due to family history | *n = 5* |
| Tested for the family variant of a pathogenic variant   - BRCA1 - BRCA2 - PALB2 - RAD51D | *n = 3*  *n = 5*  *n = 1*  *n = 1* |
| Updated genetic evaluation is pending | *n = 3* |
| Died before an updated evaluation could be performed | *n = 2* |

**Supplementary table 3:** Overview of variants of unknown significance (VUS) found in the entire cohort (left, n = 365) and the subgroup screened based on family history (right, n = 216) including number of VUS discovered in total.

| VUS discovered with NGS (all screened) (n = 80) | *APC = 3*  *ATM = 1*  *AXIN2 = 6*  *BAP1 = 1*  *BMPR1A = 2*  *BRCA1 = 6*  *BRCA2 = 17*  *BRIP1= 2 CDH1 = 2*  *CHEK2 = 1*  *DICER1 = 1*  *FANCM = 1 FLCN = 1*  *MET = 2*  *MLH1 = 2*  *MLH3 = 1*  *MSH2 = 1*  *MSH3 = 2*  *MSH6 = 2*  *MUTYH = 1*  *NF1 = 1*  *NTHL1 = 2*  *PALB2 = 4*  *PMS2 = 4*  *POLE = 5 RAD51C =3 RAD51D = 3*  *RNF43 = 1*  *TP53 = 2* | VUS discovered with NGS (familiar history) (n = 45) | *APC = 3*  *ATM = 2*  *AXIN2 = 4*  *BAP1 = 1*  *BMPR1A = 2*  *BRCA1 = 2*  *BRCA2 = 5*  *BRIP1= 1 CDH1 = 2*  *FANCM = 1*  *MET = 2*  *MLH1 = 2*  *MLH3 = 1*  *MSH2 = 1*  *MSH3 = 2*  *MUTYH = 1*  *PALB2 = 1*  *PMS2 = 2*  *POLE = 4 RAD51C =2 RAD51D = 3*  *RNF43 = 1* |
| --- | --- | --- | --- |

**Supplementary figure 1a, 1b, 1c and 1d:** Schematic view of pathogenic variants and variant of unknown significance found in the entire cohort (1a and 1c) and the subgroup screened based on family history (1b and 1d)

1a

1b

1c

1d
